# The effect of sulfasalazine in pentylenetetrazole-induced seizures in rats

**DOI:** 10.1590/1414-431X2021e11541

**Published:** 2021-12-03

**Authors:** E.S. Bora, R. Karaali, P.Y. Akyol, G. Yurtsever, O. Erbaş

**Affiliations:** 1 Department of Emergency Medicine, Izmir Katip Çelebi University Atatürk Training and Research Hospital, Izmir, Turkey; 2 Department of Physiology, Demiroğlu Bilim University Faculty of Medicine, Istanbul, Turkey

**Keywords:** Emergency service, Epilepsy, Sulfasalazine, Animal model

## Abstract

We aimed to reveal the anti-convulsant effects sulfasalazine and its mechanism in pentylenetetrazole (PTZ)-induced seizures in rats. Forty-eight male Wistar albino rats (200-250 g) were randomly divided into two groups: 24 for electroencephalography (EEG) recording (group A) and 24 for behavioral studies (group B). About 70 mg/kg PTZ was used for behavioral studies after sulfasalazine administration and 35 mg/kg PTZ was used for EEG recording after sulfasalazine administration. Electrodes were implanted on the dura mater over the left frontal cortex and the reference electrode was implanted over the cerebellum for EEG recording. Racine’s convulsion scale, first myoclonic jerk onset time, spike percentages, brain malondialdehyde (MDA), superoxide dismutase (SOD), and prostaglandin F2α (PGF2α) levels were evaluated between the groups. First myoclonic jerk onset time was significantly shorter in the saline group than both 250 and 500 mg/kg sulfasalazine groups (P<0.05). Racine's convulsion scores were significantly lower in the 250 and 500 mg/kg sulfasalazine groups than the saline group (P<0.05, P<0.001). The two sulfasalazine groups had lower spike percentages than the saline group (P<0.05). Significantly lower MDA and PGF2α levels were observed in the 250 and 500 mg/kg sulfasalazine groups compared with the saline group (P<0.05, P<0.001, respectively). SOD increased significantly in both sulfasalazine groups compared with the PTZ+saline group (P<0.05). Our study demonstrated that sulfasalazine had protective effects on PTZ-induced convulsions by protecting against oxidative and inflammatory damage associated with PTZ.

## Introduction

Neuroinflammation is a complex process that involves the activation of microglia, astrocytes, and endothelial cells in the blood-brain barrier, the infiltration of plasma proteins and immune system cells into the brain tissue, and the interaction of inflammation-related mediators with the brain tissue (1). Neuroinflammation signs are found in many central nervous system diseases, and epilepsy is often associated with neuroinflammation. Much evidence has been obtained from clinical and experimental studies that neuroinflammation increases the frequency and severity of seizures (2). Recurrent seizures are also observed in autoimmune diseases accompanied by severe and prolonged neuroinflammation and in encephalitis patients, and neuroinflammation is frequently observed in epilepsies resistant to anticonvulsant drugs (3). These findings show the importance of neuroinflammation in the pathogenesis of epilepsy and of elucidating these mechanisms for the development of antiepileptogenic therapy.

In temporal lobe epilepsy (TLE), which constitutes a large part of drug-resistant epilepsies, significant changes occur in the organization of neuronal and glial cells during the epileptogenesis process (4). The main principle within the treatment of brain disease is primarily to reduce seizures. Antiepileptic drugs (AED) treatment is successful in 65% of cases. These drugs stop seizures in two ways. The first concerns drugs like lorazepam and other benzodiazepines that reduce symptomatic seizures by increasing gaba-aminobutyric acid receptor-A (GABA-AR) mediated inhibition, and the second concerns drugs that contain phenytoin and carbamazepines, which are responsible for activating voltage-gated Na+ channels and have the effect of decreasing the potential (5-8). However, their use is limited in many patients due to their strong and intolerable side effects (9). These treatments are symptomatic and do not contribute to the basic pathological processes and diseases associated with epilepsy (10).

Increased levels of cyclooxygenase (COX) and inflammation have been reported in neurodegenerative diseases as epilepsy. COX is an enzyme that limits the metabolic rate and converts arachidonic acid into prostaglandins, which are powerful mediators of inflammation (11,12). Three COX isozymes have been identified, named COX-1, COX-2, and COX-3 (11). COX-2 is an isoform that is induced at the site of injury/inflammation and is constitutively expressed in various organs such as the central nervous system (CNS) (12). In the CNS, COX-2 is located in the dendrites of excitatory neurons, especially in the hippocampus and cerebral cortex (13). In this case, it has been suggested that COX-2 is involved in regulating important physiological processes in the brain, such as controlling body temperature, appetite, sleep, learning, and memory processes (14).

Sulfasalazine (SSZ) consists of 5-aminosalicylic acid (5-ASA) and sulfapyridine. Sulfapyridine and 5-ASA inhibit cyclooxygenase and 5-lipoxygenase. SSA is a potent and specific inhibitor of NF-κB (15). It also has antioxidant properties; it is a powerful free radical scavenger (16). SSA produces anti-inflammatory activity due to its salicylate content. It also increases adenosine deaminase levels by inhibiting purine synthesis and decreases interleukin-6 (IL-6), tumor necrosis factor-alpha (TNF-α), and IL-12 levels. SSA is thought to exert an anticonvulsant effect by inhibiting NF-kB and reducing the inflammatory process triggered by IL-1-beta (17).

With evidence of anticonvulsant effects of steroids and other anti-inflammatory therapies in patients resistant to antiepileptic drugs, speculation to the possible role of inflammation in epilepsy has emerged (18). Hence, in our study, we investigated the effects of SSZ components in the pentylenetetrazole (PTZ)-induced seizure model.

## Material and Methods

### Ethical approval

All experiments were performed in appropriate laboratories after obtaining approval from the Animal Ethics committee (Istanbul Science University, Ethics No. 05210203). All experiments were made according to the US National Institutes of Health guidelines.

### Experimental animal care

Thirty-four male Wistar albino rats weighing 200-250 g were used. Experimental animals were fed *ad libitum* and housed in steel cages with an average temperature of 22±2°C and 12-h day and night cycles that were adjusted automatically.

### Experimental procedures

For electroencephalogram (EEG) recordings (Group A) and behavioral assessment (Group B), 48 rats were randomly divided into two groups. Then, a hole was made in the skull bones with the stereotaxic method. Electrodes (polyamide-coated chrome steel wires, 0.1 mm in diameter, resistance <1Ω/10 mm) were inserted through this hole for EEG recording (1.5 mm posterior plane from lambda) (19,20). The electrodes were fixed to the skull with dental acrylic resin.

Rats were anesthetized with intraperitoneal (*ip*) ketalar (8 mg/kg) and xylazine (4 mg/kg). The kindling animal model triggered by PTZ is suitable for comparing brain activity with EEG. We used 35 mg/kg PTZ to measure epileptiform activity, but as this dose was not enough to observe behavioral changes, a dose of 70 mg/kg PTZ was used. After waiting 12 days for fixation after the electrodes were placed, we divided 24 rats into four subgroups (n=6) defined as A1, A2, A3, and A4. EEG recording is impossible with 70 mg/kg PTZ, therefore we used 35 mg/kg for recording EEG activity as previously described (19,20).

### EEG experiment (Group A)

Group A1 was non-medicated and defined as the control. Group A2 received saline intraperitoneally (*ip*), Group A3 received 250 mg/kg of SSZ (500 mg of Salazopyrin, Pfizer, USA), and Group A4 received 500 mg/kg SSZ. The drug was administered 30 min before the injection of PTZ *ip*. All groups, except group A1, received 35 mg/kg of PTZ and EEG was recorded 30 min before the PTZ injection. All protocols, behavioral analyses, and EEG graphical recordings were previously described (20,21).

EEG recordings were continued for 60 min, beginning 5 min after administration of PTZ, following the method described in the study by Erdoğan et al. (20). The signal was amplified 10,000 times and filtered in a frequency range of 1 to 60 Hz (19,20). The EEG recording was obtained using the BIOPAC MP150 data collection system (Biopac System Inc., USA). The peak level in the EEG data was evaluated by two neurophysiologists, using a revised method for assessing epileptiform activity that measured 1% of the fraction of at least one wave per conversion (20). Amplitude was at least double the baseline value, and the cumulative period of the high wave was determined at 2-min intervals (22).

### Behavioral experiment (Group B)

Following the method described by Erbaş et al. (19), 24 rats (Group B) were divided into 4 subgroups (n=6) identified as B1, B2, B3, and B4. Group B1 was non-medicated and served as the control group. Group B2 was treated with saline, and Groups B3 and B4 were treated *ip* with 250 and 500 mg/kg SSZ, respectively. Treatments were administered 30 min before the PTZ (70 mg/kg, *ip*) injection. Racine's convulsion scale (RCS) (14) and onset time of first myoclonic jerk (FMJ) were used to evaluate seizure presence and type (70 mg/kg PTZ only): 0) No seizures; 1) Twitching of vibrissae and pinnae; 2) Stiffness during exercise accompanied by clear vision; 3) Motor arrest with frequent myoclonic jerks (the timing of this stage was logged to evaluate FMJ start time); 4) Convulsive tension seizures while feeding; 5) Tonic-clonic seizure with loss of the righting reflex; and 6) Fatal seizure. Rats were assessed for FMJ onset time (TFMJ) as outlined above. Onset time was recorded in seconds. All animals showing tonic-clonic seizures died, thus observation time of seizures was limited to 30 min (23).

### Measurement of brain lipid peroxidation

Using the process described by Bora et al. (24), we used the thiobarbituric acid reactive substance (TBARS) assay to calculate malondialdehyde (MDA) levels in brain tissue samples as a measure of lipid peroxidation. Acetic acid and a TBARS chemical agent were added to the tissue samples, which then were incubated at 100°C for 60 min. After cooling in an ice bath, the samples were centrifuged at 3000 *g* for 20 min at room temperature. The absorbance was read at 535 nm and the mean absorbance was calculated. MDA levels were calculated from a standard measuring curve constructed from absorbance data for tetraethoxypropane and are reported as nmol/g macromolecule following a methodology described previously (25,26).

### Measurement of brain protein levels

The total protein concentration in brain samples was determined by Bradford's method using bovine serum albumin as a standard.

### Determination of brain SOD activity

The total superoxide dismutase (SOD) activity was determined by the method of Sun et al. (27). The principle of the method consists in inhibiting the reduction of nitro blue tetrazolium (NBT) by the xanthine oxidase system as a superoxide generator. One unit of SOD was defined as the enzyme amount causing 50% inhibition in the NBT reduction rate. SOD activity is reported as units per milligram protein (U/mg protein).

### Brain prostoglandin F2α (PGF2α) analysis

After decapitation, brains were rapidly removed and stored at −20°C until biochemical analysis. For tissue analysis, whole cerebral tissues were homogenized with a glass homogenizer in 5 vol of phosphate-buffered saline (pH, 7.4) and centrifuged at 5000 *g* for 15 min at room temperature. The supernatant was then collected and the total protein concentration in brain homogenates was determined according to the Bradford method using bovine serum albumin as a standard.

The level of PGF2α (MyBioSource Co., Ltd., USA) in the brain tissue supernatants was measured using commercially available rat enzyme-linked immunosorbent assay (ELISA) kits. All samples from each animal were measured in duplicate according to the manufacturer's guidelines. A microplate reader was used for the measurement of absorbance (MultiscanGo, Thermo Fisher Scientific Laboratory Equipment, USA).

### Statistical analysis

Results are reported as means±SE. Data analyses were performed utilizing SPSS version 15.0 for Windows (IBM, USA). Shapiro-Wilk test was used to determine if values had a normal distribution. Racine’s convulsion scores were evaluated by Kruskal Wallis test and FMJ times were evaluated by one-way analysis of variance (ANOVA). *Post hoc* Bonferonni test and Mann-Whitney U test were utilized to identify differences between the experimental groups. P<0.05 was accepted as statistically significant.

## Results

### Evaluation of groups in terms of spike percentage

**Figure 1:**
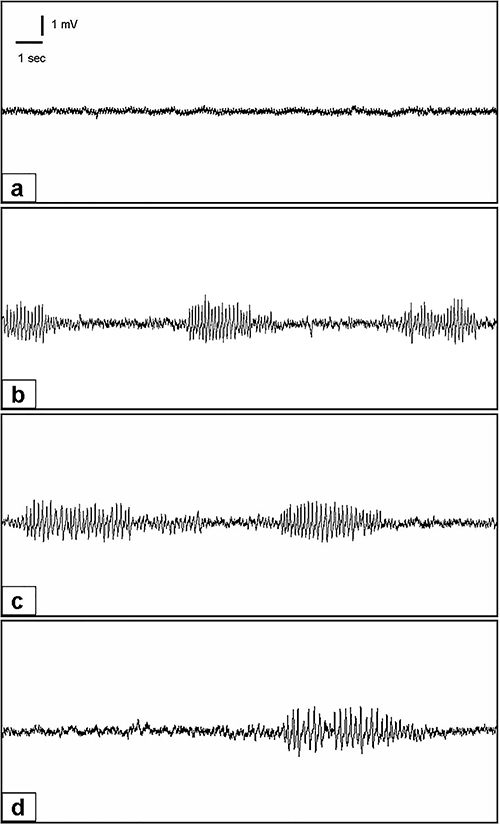
Representative EEG recordings from groups **a**: (A1, control), **b**: (A2, PTZ and saline), **c**: (A3, pentylenetetrazole (PTZ) and sulfasalazine (SSZ) at 250 mg/kg), and **d**: (A4, PTZ and SSZ at 500 mg/kg). As expected, there were virtually no spike waves seen in group A1. Dense spike wave activity was seen in group A2 due to the unopposed induction of seizures with 35 mg/kg of *ip* PTZ, with a mean spike wave percentage score of 74.6%. There was significant reduction (P<0.05) of seizure activity as quantified by spike wave percentages in groups A3 and A4 with the addition of SSZ at 250 mg/kg (A3, 48.7%) and 500 mg/kg (A4, 33.8%) compared to A2. However, the marginal improvement seen between groups A3 and A4 were not statistically different from one another.

We found that administration of 250 mg/kg SSZ significantly smothered seizure activity as measured by the percentage of spike compared to the control group (74.6 to 48.7%, P<0.05). SSZ at 500 mg/kg also suppressed seizure activity with a stronger percent efficacy (74.6 to 33.8%, P<0.05). However, there was no statistically significant difference between 250 and 500 mg/kg SSZ (Table 1). In Figure 1, high resolution information is given in order to better observe epileptiform activity in EEG.

**Table 1:** Results from the EEG experiment.

Groups	Spike percentage
A1 - Control	0
A2 - PTZ (35 mg/kg) and saline	74.6±9.5
A3 - PTZ (35 mg/kg) and 250 mg/kg sulfasalazine	48.7±11.3*
A4 - PTZ (35 mg/kg) and 500 mg/kg sulfasalazine	33.8±14.1*

Six rats were randomly distributed in each group. After sulfasalazine (SSZ) treatment, pentylenetetrazole (PTZ) at a dose of 35 mg/kg was administered to the A2, A3, and A4 groups to induce status epilepticus. The recording started 5 min after the PTZ application, and it was recorded for 60 min. Data are reported as means±SE. *P<0.05 compared to A2 (one-way ANOVA).

### Behavioral experiment results

SSZ had an antiepileptic effect in our rat model (Table 2). Compared to the control group, SSZ significantly reduced RCS and delayed TFMJ scores. The mean RCS score dropped from 5.7 (very severe as 6.0 indicates fatal seizure activity) to 2.5 (P<0.05). There was a trend towards lower RCS scores with the 500 mg/kg dose of SSZ, but this trend was not statistically significant compared with the 250 mg/kg dose of SSZ (Mann-Whitney U test). Likewise, SSZ considerably increased TFMJ at each higher dose (P<0.05). Compared to the saline-treated B2 group with an average TFMJ of 61.5 s, in the B3 group (lower dose of SSZ), the mean TFMJ was 146.3 s (P<0.05). In group B4 (higher dose of SSZ), the mean TFMJ doubled to a mean of 218.3 s (P<0.05). The difference in mean TFMJ between lower and higher SSZ doses was not statistically significant.

**Table 2:** Improvement in Racine's convulsion scale (RCS) and time of first myoclonic jerk (TFMJ) with sulfasalazine.

Groups	RCS score	TFMJ (s)
B1 - Control	0	0
B2 - PTZ (70 mg/kg) and saline	5.7±0.4	61.5±9.7
B3 - PTZ (70 mg/kg) and 250 mg/kg sulfasalazine	3.8±0.3^#^	146.3±16.2*
B4 - PTZ (70 mg/kg) and 500 mg/kg sulfasalazine	2.5±0.6**	218.3±41.3*

Rats in groups B2, B3, and B4 were given pentylenetetrazole (PTZ) at a dose of 70 mg/kg to induce apparent seizures. Data are reported as means±SE. ^#^P<0.05 compared to B2. *P<0.05, **P<0.01 compared to Control group (one-way ANOVA).

### Biochemical analysis

MDA increased from 60.7 nmol/g (control group) to 163.4 nmol/g in PTZ (70 mg/kg) + saline (P<0.05). With the administration of SSZ (250 and 500 mg/kg) after PTZ, the MDA levels decreased significantly compared with the PTZ+saline group (P<0.001). Similarly, brain SOD levels increased significantly with PTZ administration from 0.019 to 0.041 U/mg and decreased significantly with administration of SSZ (250-500 mg/kg) (P<0.05) in the PTZ+saline group. PGF2α, levels increased significantly with PTZ administration from 117.9 to 249.8 and decreased significantly with administration of SSZ (250 and 500 mg/kg) (P<0.05 and P<0.001, respectively) compared with the PTZ+saline group (Table 3).

**Table 3:** Improvement in brain malondialdehyde (MDA) level, superoxide dismutase (SOD) activity, and prostaglandin F2α (PGF2α).

Groups	Brain MDA level (nmol/g)	Brain SOD activities (U/mg protein)	PGF2α (pg/mg protein)
B1 - Control	60.7±3.9	0.019±0.08	117.9±12.1
B2 - PTZ (70 mg/kg) and saline	163.4±4.8*	0.041±0.05*	245.8±18.3**
B3 - PTZ (70 mg/kg) and 250 mg/kg sulfasalazine	92.8±6.7^##^	0.28±0.1^#^	192.2±13.4^#^
B4 - PTZ (70 mg/kg) and 500 mg/kg sulfasalazine	84.8±10.5^##^	0.25±0.09^#^	155.7±6.8^##^

Data are reported as means±SE. *P<0.05, **P<0.001 compared with the control group; ^#^P<0.05, ^##^P<0.001 compared with the B2 group (one-way ANOVA).

## Discussion

In our study, we showed that SSZ had a potential anticonvulsant effect in epileptic seizures. SSZ at 250 mg/kg reduced the spike percentage from 74.6 to 48.7% (A3 group), and 500 mg/kg SSZ reduced it to 33.8% (A4 group). Similarly, in the behavioral experiment, the TFMJ was significantly prolonged from 61.5 to 146.3 s (250 mg/kg SSZ) and 218.3 s (500 mg/kg SSZ). The convulsion stage was significantly reduced from 5.7 to 3.8 (250 mg/kg SSZ) and 2.5 (500 mg/kg SSZ). There is no previous study in the literature on the relationship of SSZ with epileptic seizures. In 2015 and 2020, Erbaş et al. (19) and Durankuş et al. (23), in their studies with dexketoprofen and ibuprofen in a PTZ epilepsy model, found that these drugs showed anticonvulsant effects in a dose-dependent manner. Doretto et al. (25) reported that dipyrone exerts anticonvulsant effects by suppressing the electrical activity in the thalamus (25); besides its antipyretic properties, dipyrone also has an anticonvulsant effect in the PTZ model. There are mixed results about the anticonvulsant effects of nonsteroidal anti-inflammatory drugs such as indomethacin. While some argue that they have antiepileptic effects through prostaglandin inhibition (26,28), others argue that it is a proconvulsant (29).

In the CNS, COX-2 is localized in excitatory neuronal dendrites (13), particularly in the hippocampus and cortex (30,31). COX-2 is involved in the regulation of important cerebral physiological processes, such as body temperature control, appetite, sleep and learning, and memory processes (14).

The effects of selective COX-2 inhibitors on the convulsions induced by PTZ are also conflicting, since selective COX-2 inhibitors have been reported to protect (32) or have no effect on the convulsions induced by PTZ (33).

Although COX-2 inhibitors such as etoricoxib decrease pro-inflammatory protein levels, it had been found that they may not lower PTZ-induced cytokine levels and stop seizures (34). This indicates that seizures cannot be prevented by COX-2 inhibition alone. In a study with a kainic (KA)-induced epilepsy model, an increase in epileptic seizures and neuronal cell death was seen before and after administration of COX-2 inhibitors. PGF2α administration at intracisterna (not PGD2 and PGE2) was found to decrease both neuronal cell mortality and epileptic seizures, but PGF2α administered without COX-2 inhibitors had little effect on epileptic seizures (35).

PGF2α values decreased in a dose-dependent manner after SSZ administration in our study. PGF2α derived from PGE2 increased in cerebrospinal fluid in children with febrile convulsion. Förstermann et al. found that PGF2α was the fastest increasing PG after PGD2 administration during seizures and this value significantly decreased after indomethacin administration (P<0.001) (35).

The study conducted by Alcoreza et al. (31) showed that SSZ plays a role in modulating glutamate levels. It has been emphasized that SSZ inhibits hyperexcitability by reducing extracellular glutamate and Mg levels. Moreover, it has a synergistic effect when used in combination with antiepileptics such as topiramate, thus it can be used with an antiepileptic or alone as an anticonvulsant.

We believe that the chronic use of SSZ could increase seizure threshold due to its anti-folate effect, as shown in the study with capecitabine (36). The SSZ component of sulfapyridine has an immunomodulatory effect similar to methotrexate, which could prevent the inflammatory process and thus the triggering of the seizures, in addition to COX-2 inhibition in the treatment of seizures. While SSZ inhibits PG synthesis by inhibition of COX-1 and -2 with its salicylate component, sulfapyridine inhibits epileptogenesis by inhibition on NFkB. Although sulfapyridine manipulates prostoglandin levels predominantly by inhibition of TNF-α, the effects of IL-6 and IL-12 should not be ignored. Inhibition of TNF-α also helps to decrease oxidative stress by the NO pathway (31,35).

Another possible relationship between inflammation and epilepsy is oxidative stress caused by inflammation in the brain, which disrupts electrical activity in neurons and causes epileptic spasms. Oxidative stress increased considerably in the PTZ and saline group, whereas an important decrease was shown in the PTZ-SSZ group (250 and 500 mg/kg). Oxygen free radicals generated by nicotinamide adenine dinucleotide phosphate (NADPH) as a result of oxidative stress are known to disrupt the electrical balance and thus lower the epilepsy threshold (31). In addition to inflammatory suppression and COX inhibition by SSZ, suppressing post-seizure free radicals also limits neuron damage.

One of the study’s limitations is that we did not use a group with a classical antiepileptic drug as a control group because of the lack of rats. On the other hand, the literature indicates that the toxic dose is 600 mg/kg and we did not aim to evaluate the toxic effects of SSZ in this study.

### Conclusions

This study demonstrated the effect of SSZ on convulsions through behavioral assessment, EEG, and PGF2α level in the brain. It can be used safely for epileptic seizures, and it may be used with AED for synergic effects. However, more experimental and clinical studies are needed.
